# New minimally invasive technique for repositioning of a subluxated retropupillary iris-claw IOL using needle-assisted fixation

**DOI:** 10.1186/s40942-025-00651-y

**Published:** 2025-03-10

**Authors:** Celso Costa, Nuno Gouveia, Pedro Fonseca, Miguel Raimundo, Joaquim Murta, João Figueira

**Affiliations:** 1 Department of Ophthalmology, Unidade Local de Saúde de Coimbra (ULS Coimbra), Coimbra, Portugal; 2 Clinical Academic Center of Coimbra (CAAC), Coimbra, Portugal; 3 Faculdade de Medicina da Universidade de Coimbra (FMUC), Coimbra, Portugal; 4 Unidade de Oftalmologia de Coimbra (UOC), Coimbra, Portugal

**Keywords:** Subluxation, Retropupillary iris-claw IOL, Needle-assisted

## Abstract

**Purpose:**

This paper presents a modification of the needle-assisted retropupillary fixation technique for iris-claw intraocular lenses (IOLs). We introduce a novel, minimally invasive reenclavation technique for managing subluxated retropupillary iris-claw IOLs.

**Methods:**

The technique was successfully performed on four patients diagnosed with subluxated retropupillary iris-claw IOLs. This approach involved a novel, minimally invasive reenclavation technique that utilized three needles: one long, straight translimbal needle crossing the anterior chamber and two needles introduced through the pars plana to reposition and securely reenclavate the IOL.

**Results:**

All patients experienced immediate and significant visual recovery, with their baseline visual acuities reestablished postoperatively. Importantly, no post-operative complications were observed during the six-month follow-up period, highlighting the safety and efficacy of the technique.

**Conclusion:**

Our novel, minimally invasive technique for reenclavation of iris-claw IOLs, employing just three needles, eliminates the need for trocar insertion, infusion cannulas, or sutures. This approach offers significant advantages, including a shorter operative time and minimal surgical manipulation, leading to faster patient recovery and fewer complications.

**Supplementary Information:**

The online version contains supplementary material available at 10.1186/s40942-025-00651-y.

## Background and purpose

The surgical management of aphakia without capsular support remains challenging. Two primary techniques are commonly employed: scleral fixation of a posterior chamber intraocular lens (SF-PCIOL), either with or without sutures, and iris-claw intraocular lenses (IC-IOL) [1, 2]. More recently, the fixation of IC-IOLs to the posterior iris surface has been introduced [3], offering several advantages. These include positioning behind the iris, away from the corneal endothelium, reduced complications, and improved visual outcomes [4, 5], though this comes with increased technical complexity. The technique can involve using an implantation forceps to hold the center of the IOL, coupled with an enclavation needle that enters through paracentesis at the 2 and 10 o’clock positions to perform the enclavation of the Artisan. Prieto et al. in 2017 [6] described the needle-assisted retropupillary fixation of iris-claw intraocular lenses as an alternative.

This technique begins with the passage of a long straight needle from a 10/0 Prolene suture through the corneal limbus, starting 1 mm behind it, from the 9 o’clock to the 3 o’clock position, crossing the anterior chamber. Using lens forceps, the iris-claw IOL is introduced through a 5.4 mm corneal incision and rotated into the horizontal plane, with its concavity facing the endothelium. The IOL is then positioned behind the iris and tilted towards the posterior iris surface. The anterior chamber needle serves as a pivot to enclave the haptics. Finally, the long straight needle is carefully removed.

There is limited information available in the literature regarding the management of subluxated retropupillary IC-IOLs, a complication that is not uncommon [7], with reported rates ranging from 0 to 37% [8]. Surgical approaches include combinations of full 3-port vitrectomy [9] or an anterior chamber maintainer with the creation of a scleral tunnel or a paracentesis [10]. These more invasive techniques have the disadvantages of increased operating time and cost. We describe a novel, minimally invasive reenclavation technique for managing subluxated retropupillary iris-claw intraocular lenses.

### Surgical technique

**Fig. 1:**
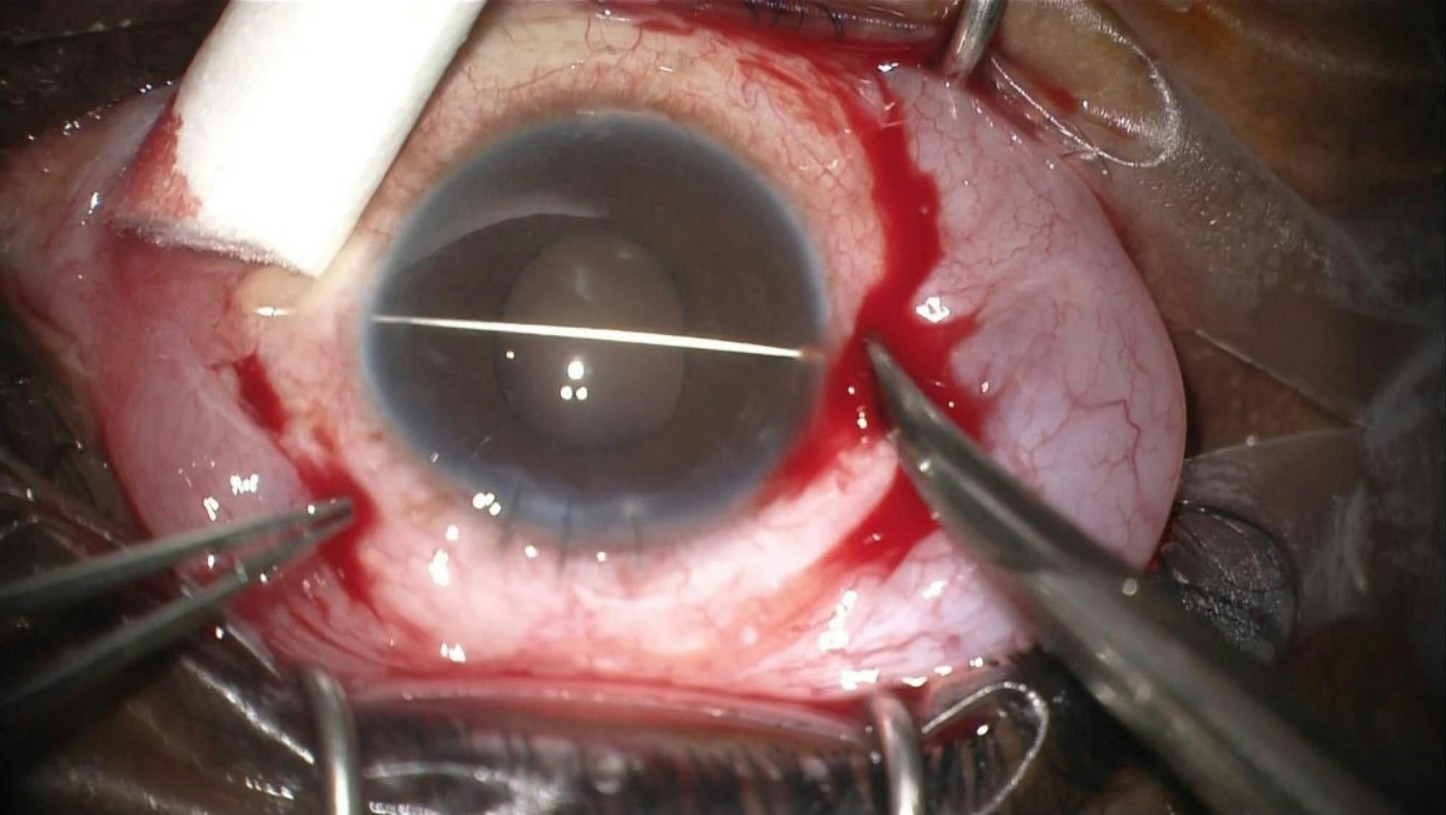
The long straight unarmed needle of a 10/0 prolene is introduced across the anterior chamber

**Fig. 2:**
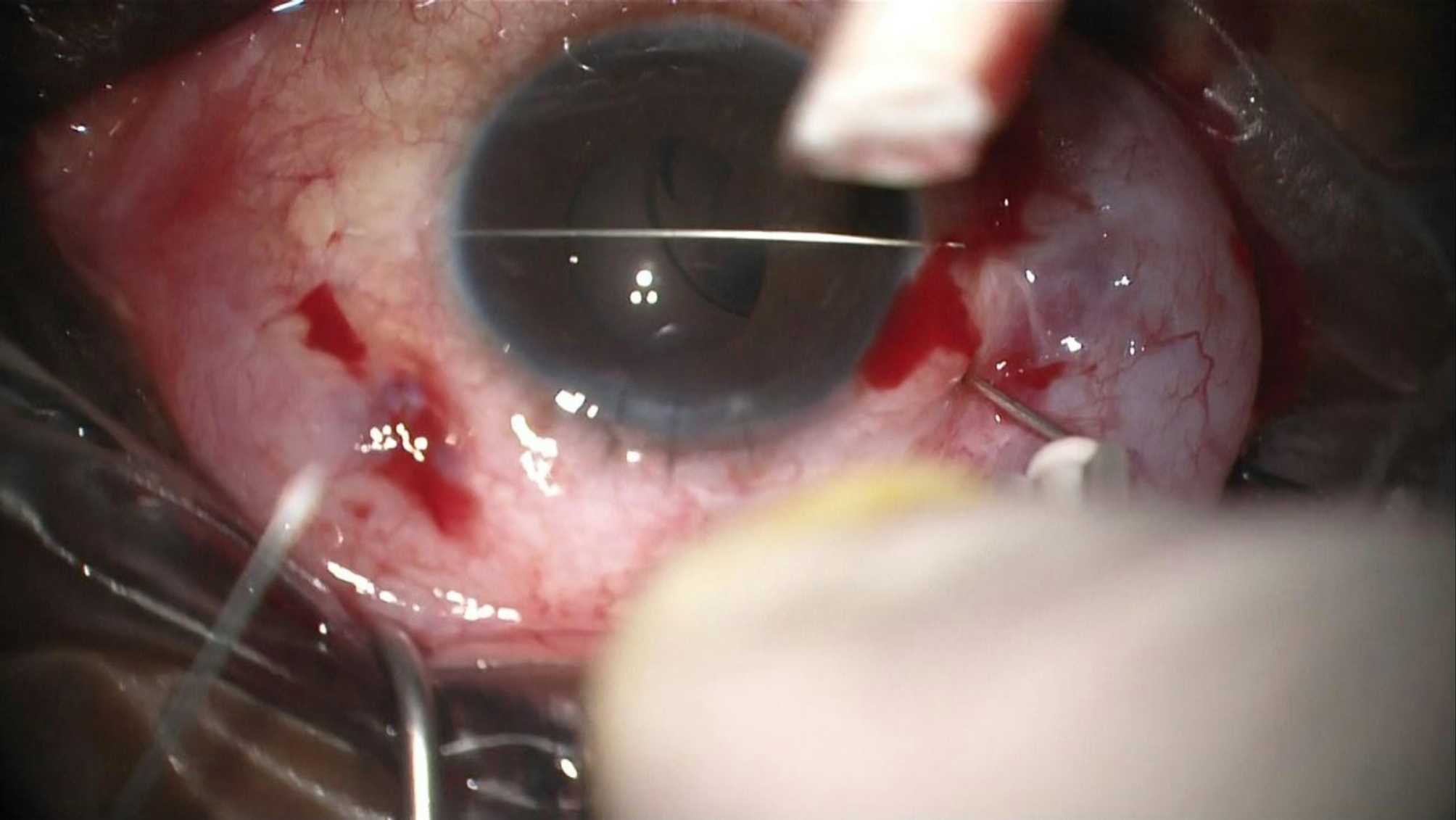
Two 27-gauge needles bent at a 45 degrees angle are introduced through the pars plana across the anterior vitreous cavity, moving the IOL to the correct plane

The technique involves a minimally invasive reenclavation approach using three needles. First, a long straight 0.2 Ph.Eur. STC-6 16 mm reverse cutting needle from a 10/0 Prolene suture is introduced across the anterior chamber, as previously described (Fig. 1). Next, two 27-gauge needles, bent at a 45-degree angle, are introduced through the pars plana, preferably laterally and superiorly (Fig. 2). These needles are used to push each haptic anteriorly towards the posterior iris surface, employing coordinated movements of both hands.

**Fig. 3:**
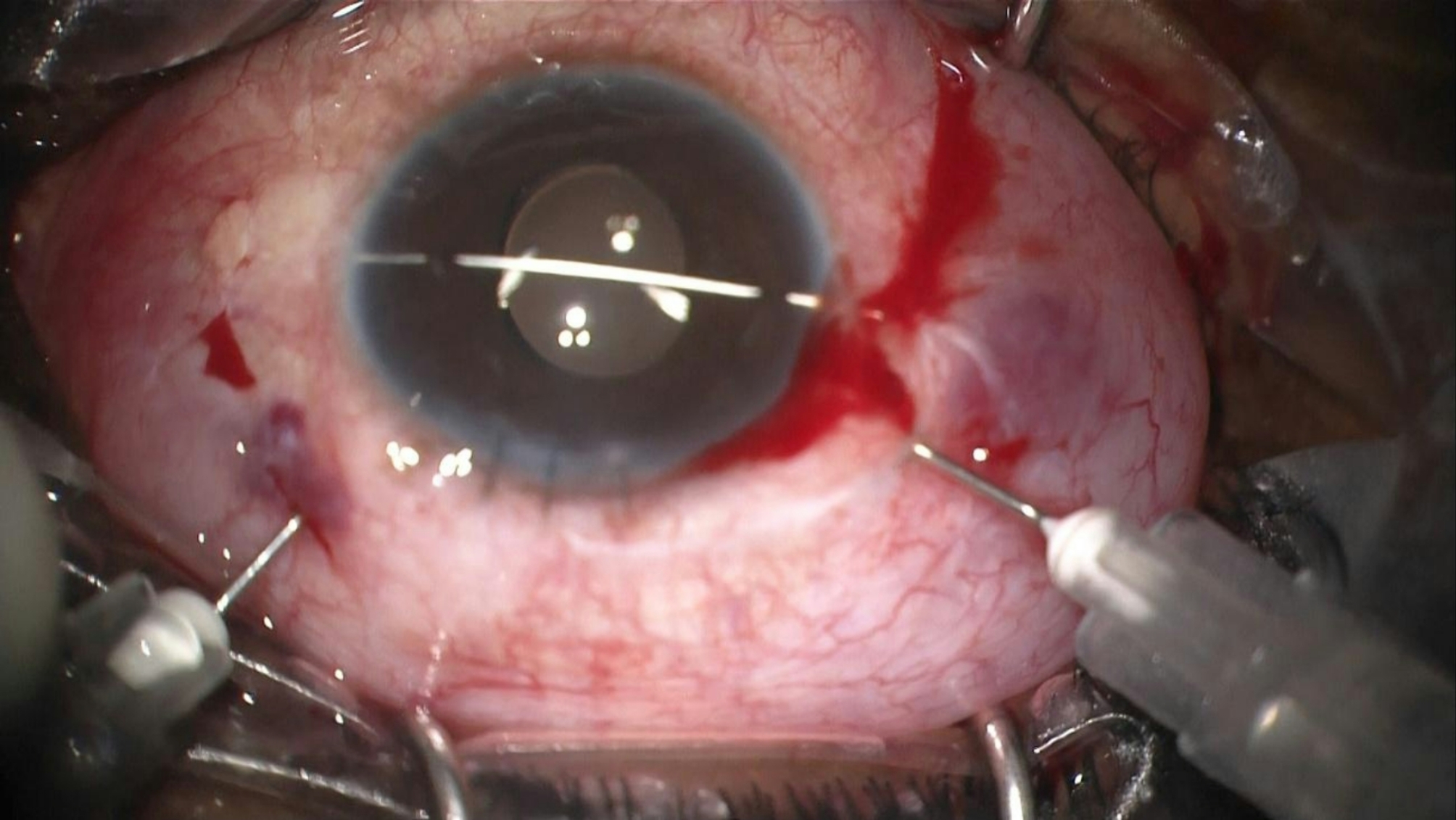
Enclavation of the IC-IOL using counter-action from the anterior chamber needle

**Fig. 4:**
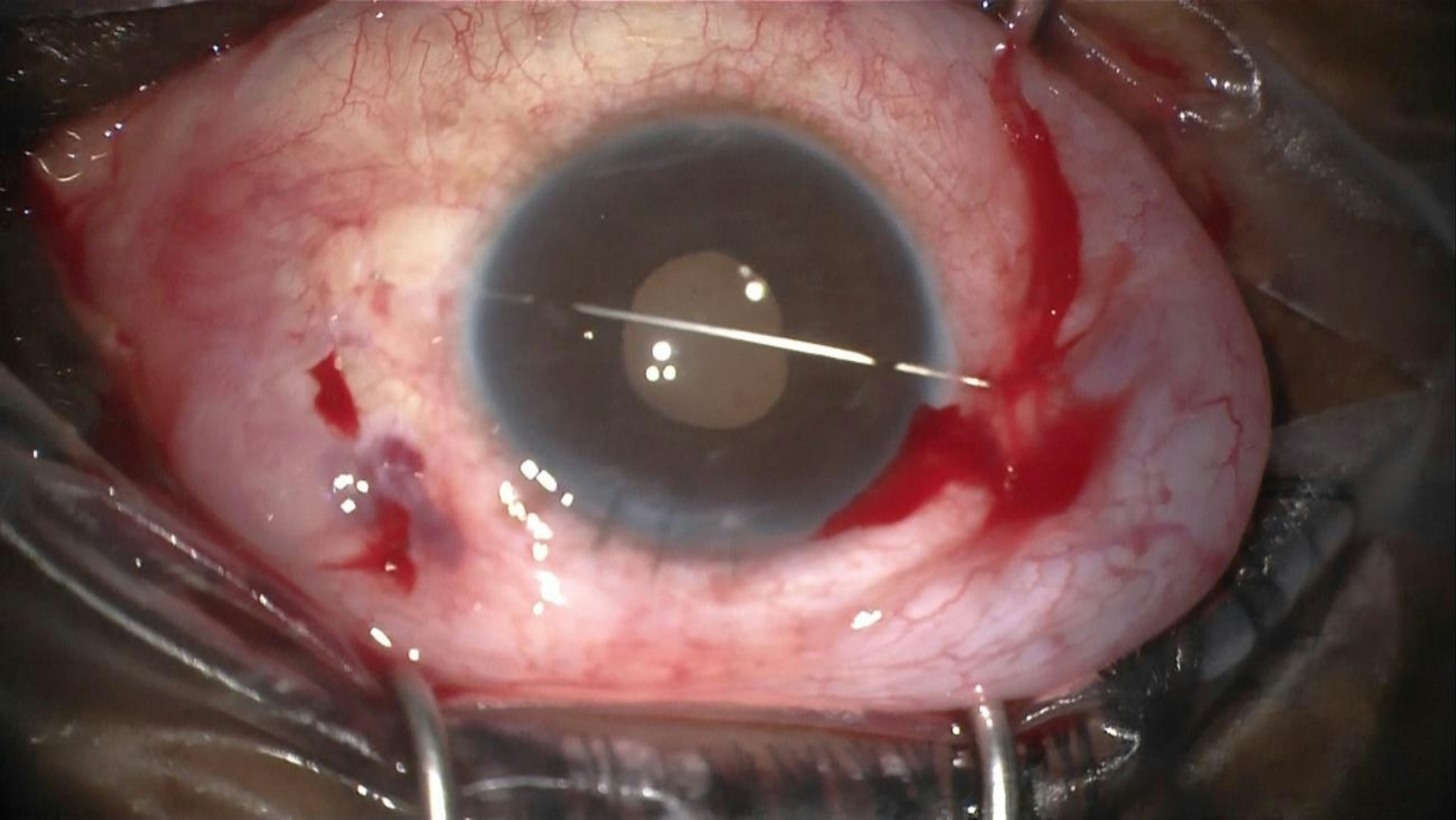
The long straight needle functioning as a pivot to enclave both haptics, before removal

**Fig. 5:**
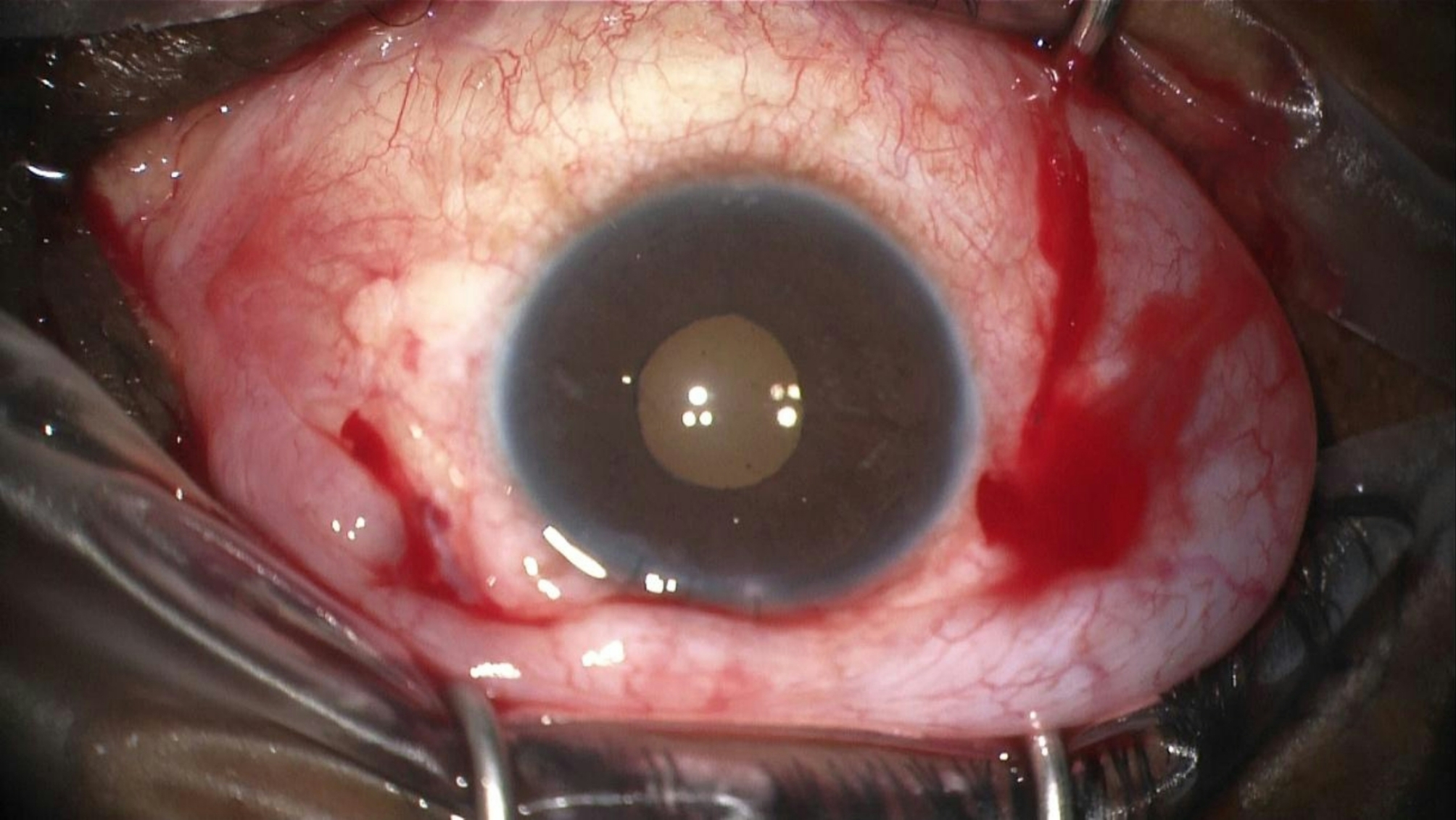
After removing the anterior chamber needle, the IOL remains stable and the pupil round

The first needle is passed below the IOL at the point where the haptic is enclavated to the iris. This needle elevates the IOL and holds it in position while the other needle enclavates the iris within the haptic on the opposite side. This maneuver ensures correct positioning and stabilization of the IOL, facilitating the enclavation of the haptic against the anterior chamber needle (Fig. 3). Finally, the straight needle is removed, leaving the IOL in situ and maintaining a round and regular pupil (Figs. 4 and 5).

### Patient characteristics and surgical outcomes

We conducted a retrospective, non-comparative consecutive case series study involving four patients (four eyes) treated at our institution between May 2022 and May 2023. These patients presented with spontaneous or traumatic single-haptic subluxated retropupillary iris-claw IOL (Ophtec Artisan Aphakia). Informed consent was obtained from all participants, and the study adhered to the principles outlined in the Declaration of Helsinki for biomedical research. Surgical procedures were performed by two experienced surgeons (MR and JF). Table 1 provides details of the clinical characteristics and outcomes of the patients.

The representative case (Figs. 1, 2, 3, 4 and 5) involves Patient 1, an 85-year-old male who presented to our department with a history of blunt trauma to the left eye two days prior. The patient had undergone cataract surgery in the left eye elsewhere 15 years ago, which resulted in posterior capsule rupture and subsequent extensive anterior vitrectomy and retropupillary artisan aphakia IOL implantation. On examination, his visual acuity was counting fingers without correction, improving to 20/25 with + 9.00D correction. Intraocular pressure was 21 mmHg, and slit-lamp examination revealed a single-haptic dislocation of the retropupillary iris-claw IOL. Fundoscopic examination was unremarkable. Following surgery, the patient experienced an immediate recovery, with visual acuity improving to 20/25 without correction and 20/20 with − 0.50 × 90º refraction. There were no postoperative complications, and the IOL was well-centered. At the 6-month follow-up, the IOL remained well-positioned. A video demonstrating the surgical technique is available as a digital supplement to this manuscript (see Video, Supplemental Digital Content 1).

In our series, only one patient experienced recurrence of the subluxation (Patient 2). He had a history of ocular trauma 10 years ago, followed by traumatic cataract surgery, IOL implantation in the capsular bag, and pars plana posterior vitrectomy due to inferior macula-sparing rhegmatogenous retinal detachment. Subsequently, the retina redetached, and the IOL dislocated. He underwent a new vitrectomy, and the IOL was exchanged for a three-piece IOL in the ciliary sulcus. This sulcus IOL also subluxated, necessitating explantation and retropupillary implantation of an iris-claw IOL. Six months later, one of the haptics of the IOL disengaged from the iris, requiring reenclavation as described in this manuscript (see Video, Supplemental Digital Content 2). Months later, another haptic disengaged from the iris, leading to the decision to fixate the subluxated IOL to the anterior iris surface (with its concavity facing the posterior segment). Upon examination, we discovered that the subluxated haptic had a defect, likely contributing to the repeated disenclavations. With this anterior approach we were able to enclave the haptics with much more iris tissue than with the retropupillary approach, so the IOL is stable, as we are able to prove through direct observation.

Patient 3 had a history of IOL-capsular bag complex subluxation years after cataract surgery. We decided to explant the complex and implant a retropupillary IC-IOL, which subsequently subluxated in the following months. We performed our technique and managed this case successfully.

Patient 4 had a history of ocular trauma with an intraocular metallic foreign body that required immediate surgical intervention. She remained aphakic and used a rigid contact lens for 18 years. More recently, she developed intolerance to the contact lens, prompting us to implant a retropupillary IC-IOL, which subluxated two months later during a dental procedure involving a vibrating drill. This case was successfully managed as described in this report.

**Table 1:** Clinical results of patients submitted to minimally invasive retropupillary iris-claw intraocular lens using needle-assisted fixation

	Patient 1	Patient 2	Patient 3	Patient 4
Sex/age (years)	M/85	M/44	M/83	F/73
Pre-operative				
UCVA	CF	HM	CF	CF
BCVA (aphakic)	20/25	20/40	20/32	20/40
IOP	21	14	13	15
Pseudoexfoliation	No	No	Yes	No
Subluxation (spontaneous vs. traumatic)	Traumatic	Spontaneous	Spontaneous	Traumatic
Post-operative (6 months)				
UCVA	20/25	20/50	20/32	20/40
BCVA	20/20	20/40	20/25	20/25
IOP	15	16	12	15
Subluxation recurrence	No	Yes	No	No

BCVA, best-corrected visual acuity; UVCA, uncorrected visual acuity; IOP, intraocular pressure; HM: hand motion; CF, counting fingers

## Discussion and conclusions

The anterior and retropupillary fixation of an iris-claw IOL provides effective options for managing aphakia in the absence of capsular support. According to Mora et al. [5], fixating the IOL behind the iris yields visual outcomes and postoperative complications comparable to anterior IOL fixation. However, Al-Dwairi et al. [4] reported even better outcomes with retropupillary fixation, including reduced IOL decentration or tilt, less postoperative intraocular pressure elevation, lower incidence of macular edema, and improved visual acuity.

The needle-assisted technique simplifies the enclavation process compared to the classic microspatula method [11]. This technique reduces the number of attempts needed to capture the iris, minimizing pigment dispersion and iatrogenic iris damage while promoting a more natural, round pupil shape. Additionally, by ensuring that fixation points are directly opposite and equidistant from the pupil, this approach reduces iris tissue stretching, resulting in less pupil ovalization.

Single-haptic dislocation of a retropupillary IC-IOL may be more common than previously recognized, and the optimal management strategy remains a subject of debate. Our group first performed this technique in 2022, achieving favorable outcomes [12]. Another variation involves the use of two 30-gauge needles, one connected to a viscosurgical device [8]. In our experience, utilizing a single needle behind the IC-IOL complicates the process of correctly positioning, stabilizing, and refixating the lens.

Regarding disenclavation, we hypothesize that in some patients, the iris may become significantly fibrotic. Since it is impossible to directly evaluate the posterior surface of the iris, a fibrotic layer might be present. In such cases, recurrent episodes of disenclavation could occur. Therefore, it is recommended to redo the enclavation, securing the haptic with the use of a thread. Mora et al. [13] considered the presence of any sign of iris atrophy an exclusion criteria in their paper regarding light- and drug-induced pupillary dynamics in eyes with a retropupillary iris-claw intraocular lens, which supports our hypothesis.

Our technique can be performed under local or sub-Tenon anesthesia. This novel, minimally invasive reenclavation method, which requires only three needles, eliminates the need for trocar insertion, infusion cannula, scleral tunnels, corneal paracentesis, viscoelastic, or sutures. Although often unnecessary, the use of an infusion trocar or an anterior chamber maintainer could be considered by surgeons when operating on eyes that have undergone a posterior vitrectomy to maintain stable intraoperative eye pressure, as there may be an increased risk of intraoperative hypotony, especially if difficulties are encountered during the initial attempts at IOL enclavation. The short operative time, reduced surgical costs, and minimal surgical manipulations of our technique make it a relatively straightforward procedure. Additionally, patients experience rapid visual recovery, which is crucial given that these patients often undergo multiple surgeries and require simple yet effective interventions. Anterior vitrectomy is a prerequisite, considering the risk of vitreous traction in non-vitrectomized eyes.

The limitations of our study are inherent to a case series study, as the limited sample size may affect the generalizability of the results. Given the advantages and low complication rates associated with this technique, we advocate for its broader adoption, as well as conducting studies with a larger cohort of patients. This will be essential to identify any potential complications associated with the technique and robustly validate the outcomes.

## Electronic supplementary material

Below is the link to the electronic supplementary material.

Supplementary Material 1

Supplementary Material 2

## Data Availability

No datasets were generated or analysed during the current study.

## Declarations

### Financing support

This work has not received any contribution, grant or scholarship.

### Confidentiality of data

The authors declare that they have followed the protocols of their work center on the publication of data from patients.

### Protection of human and animal subjects

The authors declare that the procedures followed were in accord-ance with the regulations of the relevant clinical research ethics committee and with those of the Code of Ethics of the World Medical Association (Declaration of Helsinki as revised in 2013).

### Provenance and peer review

Not commissioned; externally peer reviewed.
